# Nucleolar and Ribosomal Dysfunction—A Common Pathomechanism in Childhood Progerias?

**DOI:** 10.3390/cells8060534

**Published:** 2019-06-04

**Authors:** Tamara Phan, Fatima Khalid, Sebastian Iben

**Affiliations:** Department of Dermatology, Ulm University, James-Franck Ring N27, 89081 Ulm, Germany; tamara.phan@uni-ulm.de (T.P.); fatima.khalid@uni-ulm.de (F.K.)

**Keywords:** nucleolus, aging, Hutchinson–Gilford Progeria syndrome, Cockayne syndrome, trichothiodystrophy, RNA polymerase I, ribosome, translational fidelity

## Abstract

The nucleolus organizes around the sites of transcription by RNA polymerase I (RNA Pol I). rDNA transcription by this enzyme is the key step of ribosome biogenesis and most of the assembly and maturation processes of the ribosome occur co-transcriptionally. Therefore, disturbances in rRNA transcription and processing translate to ribosomal malfunction. Nucleolar malfunction has recently been described in the classical progeria of childhood, Hutchinson–Gilford syndrome (HGPS), which is characterized by severe signs of premature aging, including atherosclerosis, alopecia, and osteoporosis. A deregulated ribosomal biogenesis with enlarged nucleoli is not only characteristic for HGPS patients, but it is also found in the fibroblasts of “normal” aging individuals. Cockayne syndrome (CS) is also characterized by signs of premature aging, including the loss of subcutaneous fat, alopecia, and cataracts. It has been shown that all genes in which a mutation causes CS, are involved in rDNA transcription by RNA Pol I. A disturbed ribosomal biogenesis affects mitochondria and translates into ribosomes with a reduced translational fidelity that causes endoplasmic reticulum (ER) stress and apoptosis. Therefore, it is speculated that disease-causing disturbances in the process of ribosomal biogenesis may be more common than hitherto anticipated.

## 1. Ageing and Progeria

Ageing is a complex and unavoidable process that can be defined as the functional decline after a period of maturity. In this respect, maturity is understood as the endpoint of development and the condition of maximal functional performance capability. Aging is a dynamic process, as is maturity—biological systems in the form of cells and multicellular organisms do not reach a defined condition of maturity. By comparison, development results in states of maximal functional performance capability of subcellular signaling pathways or individual tissue functions, whereas other, adjacent pathways or tissues are already in decline. For example, humans reach their maximal sprint velocity at the age of 23 years (Usain Bolt, World Record 2008), but their maximal endurance capability ten years later (Triathlon, Hawaii), when they have already lost maximal speed. When the maximal creative and intellectual performance of the human mind is reached, is currently not precisely defined [1]. This heterogeneity of aging is reflected in multiple different pathways that all result in functional impairment and are defined as hallmarks of aging [2]. 

Frequently, these hallmarks are interrelated, for example, telomere attrition as one hallmark leads to genomic instability, another hallmark. This association is typical for complex systems, such as biological organisms that are dynamically interrelated as though in a four-dimensional network. 

Because aging predisposes to age-associated disease and is the primary risk factor for diseases like cancer, dementia, and osteoporosis, it is of utmost importance for an aging society to know how to prevent and treat age-associated diseases. To study the aging process and the underlying biological pathways in humans, one can either investigate exceptional human longevity, centenarians who have a deceleration of morbidity, or the opposite situation, where children age as if in time lapse and suffer from accelerated morbidity. These childhood syndromes, termed progerias, are of segmental nature, in that they display some symptoms of aging without mirroring the whole spectrum of age-associated disease. These “caricatures of aging” are also denominated as premature aging, in which aging occurs before entering the state of maturity. This also implies that the deleterious forces that drive the aging processes in these children are stronger than the opposing forces of growth and development to maturity. Therefore, it is very interesting to understand these forces, the pathomechanisms of premature aging, as they will help to understand the pathomechanism of the aging process itself. Premature aging syndromes serve as model diseases to study aging, although not all of them have been validated as showing a pathomechanism that also drives the “normal” aging process, and therefore numerous unresolved questions remain to be answered. 

The progeria of the adult, Werner syndrome, is also characterized by an accelerated, segmental aging process that starts with a lack of the pubertal growth spurt. In the second and third decade it develops with a loss of subcutaneous fat and signs of skin aging, cataracts, hair loss, atherosclerosis, and osteoporosis [3]. Werner syndrome patients suffer from a broad spectrum of age-associated diseases, but do not show the age-associated neurodegenerative diseases like Alzheimer or Parkinson dementia. Most cases of Werner syndrome are caused by mutations in the WRN-helicase, involved in multiple DNA metabolic pathways such as DNA transcription, replication, repair, and telomere maintenance and the affected cellular pathways cover the whole spectrum of the molecular hallmarks of aging [2,4,5]. Werner syndrome fibroblasts display a severe reduction of replicative lifespan in culture and enter senescence after few passages [6]. Accumulation of senescent cells is one driver of the aging process in humans [7], however, it became clear in the case of Werner syndrome and the pleiotropic WRN-helicase that it is not possible to decipher the critical function whose failure causes accelerated aging and age-associated diseases. Therefore, the recent discussions emphasize the pleiotropic nature of the aging process in Werner´s syndrome [4,5]. Nonetheless, investigating progerias is one opportunity to analyze if there are single cellular pathways existing that are critical for the rate of aging in humans. Due to the fact that progerias are caused by known and defined mutations and their segmental nature it should be possible to identify if there are particular cellular pathways involved in, or responsible for the accelerated aging process. 

In this review we would like to address if, and how, the nucleolus and RNA polymerase I transcription might contribute to the segmental aging pathologies of childhood progerias.

## 2. Aging and the Nucleolus

One question is whether the nucleolus, the site of ribosomal transcription and synthesis, is involved in the aging pathomechanisms or even a driver of age-associated physiological decline. The nucleolus is a non-membrane-bound organelle that organizes around the sites of rDNA transcription by RNA polymerase I (RNA Pol I) and the co-transcriptional pre-ribosomal assembly and processing [8]. The nucleolus is a dynamic organelle that dis- and reassembles during the cell cycle and contains a multitude of proteins involved in ribosomal biogenesis, cell cycle regulation, and stress responses [9,10]. 

In yeast cells, it has been shown that the redistribution of the silence information regulator, Sir2, from the telomeres to an AGE locus prolongs lifespan. The nucleolus has been identified as the AGE locus [11] and the accumulation of extrachromosomal rDNA circles resulting in nucleolar enlargement and fragmentation is one cause of aging in yeast [12]. This aging mechanism could not be confirmed in mammals, however the nucleolus attracted the attention of the aging research community. 

The size of the nucleolus, during early microscopy, was recognized as, and is still valid as, a prognostic factor in tumor pathology and an indicator of the activity of the ribosomal synthesis machinery [13]. The nucleolar size, the activity of ribosomal biogenesis, and subsequent protein synthesis inversely correlate with lifespan in model organisms like *Caenorhabditis elegans* [14]. This implies that individuals with a high protein synthesis rate and turnover as well as large nucleoli might age more rapidly than individuals with a slow ribosome synthesis/protein synthesis activity and small nucleoli. This observation is further supported by the fact that inhibition of a central sensor/regulator of nutrient availability, mammalian target of rapamycin (mTOR), also regulates nucleolar size and activity and inhibition of mTOR by rapamycin which results in a reduced nucleolar size, alleviates age-related pathology, and even extends the lifespan of model organisms [15]. The metabolic theory of aging states that the metabolic rate of an organism, which is also mirrored in nucleolar size and activity, is one denominator of the aging process and lifespan [16,17]. Therefore, the nucleolar size and activity reflect metabolic conditions, and thereby the rate of cell and tissue aging, however, whether this is mere correlation or a causal association is currently not established. Consequently, the investigation of the pathomechanisms of progerias may help to determine whether the nucleolus is a downstream effector or a causal player in the chain of events that leads to the demise of the cellular, tissue, and organismal integrity during the aging process.

## 3. Nucleolar Size as a Hallmark of Aging

A study in *C. elegans* by the Antebi group [18] identified the nucleolus as a central effector in longevity pathways. Asking whether all lifespan-extending signaling pathways converge on the same downstream process, they discovered that nucleolar size and function are predictive markers of life expectancy. Starting from the identification of nucleolin (NCL1) as a downstream effector of caloric-restriction mediated lifespan extension, they demonstrated that NCL1 knockdown abrogated all the tested life-extending manipulations. NCL1 is a protein that inhibits rRNA transcription and protein synthesis [19]. Knockdown of *ncl-1* in *C. elegans* led to enlarged nucleoli in several tissues [20]. Knockdown of *ncl-1* blocked the lifespan-extending effects of the manipulations of a genetic caloric-restriction model on the TOR pathway, the insulin/insulin-like growth factor pathway, the germline-less mutants, the mitochondrial effectors, the translation-initiation factors, and the ribosomal S6kinase. Overexpression of *ncl-1* reduced nucleolar size and increased lifespan. All lifespan-extending models exhibited small nucleoli and the size of the nucleoli in wild-type *C. elegans* was a predictor of life expectancy. Reduced nucleolar size was associated with a reduced ribosomal RNA and protein content, suggesting that the downregulation of ribosomal synthesis is an indicator of long life. Finally, the authors analyzed nucleolar size in long-lived *Drosophila melanogaster* and observed reduced nucleolar size in these insects as well as in muscle biopsies of dietary-restricted, exercising humans. This study, thereby, identified nucleolin and its nucleolar function as a central player in lifespan regulation and is in agreement with the metabolic theory of aging which proposes that interventions that dampen high-energy demanding processes, including ribosomal biogenesis and translation, prolong the lifespan. Moreover, the findings that small nucleoli are related to longevity [18] and large nucleoli to premature aging [21], could, when confirmed in larger human cohorts, be used as predictive parameters of metabolic health, biological aging, and life expectancy. 

## 4. Hutchinson–Gilford Progeria Syndrome (HGPS)

The HGPS, frequently termed childhood progeria, is a severe developmental and degenerative syndrome that is characterized by a typical facial appearance (bird-like facies) with micrognathia, alopecia, and prominent veins on the forehead. Hypoplasia of the claviculae, generalized osteoporosis with pathological fractures, and the lifespan-limiting general atherosclerosis are hallmarks of this syndrome. Life expectancy is severely reduced because of cardiovascular insults, including stroke and heart infarction in the second and third decades (mean life expectancy of 14.6 years) [22]. HGPS is a segmental progeroid syndrome, because it does not mirror the full spectrum of age-associated diseases. Neurological degeneration and dementia are not found in these children. Most cases of HGPS result from a dominant de-novo mutation in the germline causing the activation of a cryptic splice site in the lamin A/C gene. This is followed by a miss-splicing that removes parts of exon 11, resulting in the loss of 50 amino-acid sequence within the protein. These 50 amino acids in lamin A/C comprise a cleavage site that is essential for lamin A maturation. As a consequence, immature lamin A with a noncleaved farnesyl group, termed progerin, accumulates in the cells and integrates into the nuclear lamina [23]. HGPS is associated with significant changes in nuclear shape, including lobulation of the nuclear envelope, thickening of the nuclear lamina, loss of peripheral heterochromatin, and clustering of nuclear pores [24]. Progerin, the misprocessed immature lamin A, has been found to accumulate during the normal aging process [25], thus confirming HGPS on the molecular level as a progeroid disease of accelerated aging. The investigation of nuclear changes in HGPS have enhanced our knowledge about the identation of the nuclear envelope with nuclear processes, including chromosomal territory organization, chromatin regulation, transcription, DNA repair, and mechanosignaling [22,26,27,28]. 

Considering progerin and if it influences the proteome of HGPS cells, Buchwalter and Hetzer discovered that nucleolar dysregulation might be involved in the pathophysiology of premature aging [21]. By labelling newly synthesized proteins and analyzing the nuclear proteome, they found that elevated translation produced more proteins in HGPS cells than in control cells. A greater proportion of ribosomes were engaged in translation as compared with parental control cells. The HGPS cells were found to cycle more rapidly than wild-type cells, indicating that through this mechanism the HGPS cells can cope with the higher protein load. Because protein synthesis is regulated by the mammalian target of rapamycin (mTOR) pathway, the authors analyzed the signaling and could not find an elevated signal transduction cascade of mTOR, however did find an increased level of the ribosomal subunit RPS6. The nucleoli were enlarged and produced more pre-rRNA precursor, resulting in elevated levels of mature 28S and 18S rRNA. This was found to be due to a hypo-methylation of the rDNA promoter in HGPS cells, enabling the engagement of the RNA Pol I on more templates. Concomitantly, the amount of nuclear and cytosolic ribosomal proteins was found to be elevated in HGPS cells. To establish a causal link between the progerin expression and the altered nucleolar architecture, the authors expressed progerin and control lamin A, and monitored nucleolar expansion. They demonstrated that progerin inhibits the nucleoplasmic distribution of lamin A and that nucleoplasmic lamin A restricts nucleolar size and activity. These findings delineate an unexpected association between the nuclear lamina, nucleolar organization, and regulation of protein synthesis output. Reducing protein translation with translation-inhibiting drugs or by knockdown of ribosomal proteins can extend the lifespan of *C. elegans* and mice [29,30]. Asking whether an enhanced or deregulated nucleolar activity is a hallmark of aging, Buchwalter and Hetzer compared nucleolar size and rRNA content of human fibroblasts from young and old donors. They found an increase in nucleolar size and the rRNA content with age, suggesting that an increase in progerin expression [25] and a heterochromatin loss [31] in elderly subjects might result in enhanced ribosomal synthesis, translational activity, and protein synthesis. Therefore, one hallmark of aging, loss of proteostasis [2], might be mediated by a deregulated protein synthesis originating from an accumulation of progerin. In the Buchwalter and Hetzer study, the authors identified the nucleolus and its size and activity, for the first time, as a central player in the pathophysiology of HGPS. It remains to be investigated whether inhibitors of a deregulated ribosomal biogenesis, such as CX5461 [32], might influence disease progression and survival in mouse models of HGPS [33]. 

## 5. Cockayne Syndrome (CS)

The CS is a rare autosomal recessive disease that can be caused by six different genes, which are all involved in nucleotide excision repair (NER) of UV lesions [34]. It is characterized by a high skin UV sensitivity and severe developmental and degenerative disturbances with a mean life expectancy of 12 years [35,36]. CS patients present with a failure to thrive and, depending on the severity, degeneration of different organ systems, including the loss of subcutaneous fat tissue (“cachectic dwarfs”), alopecia, cataracts, and neurodegeneration. Neurodegeneration affects the peripheral system (hearing loss) as well as the central nervous system, with intellectual, gait, and speech impairments. Most of the symptoms found in this syndrome are normally associated with advanced age (cachexia, alopecia, cataracts, neurodegeneration), thus the CS presents as a progeroid disease. The CS is of segmental nature, because it does not display an elevated cancer incidence that is typical for xeroderma pigmentosum (XP), a high cancer-prone skin disease that is also caused by mutations in NER genes. Because mutations in the NER factors, which completely inactivate NER, are not followed by childhood degeneration but rather by XP, which is not characterized by a severe developmental failure [37], alternative explanations for the pathogenesis of premature aging are discussed [38]. One particular cellular feature that distinguishes the CS cells from cells of non-progeroid NER syndromes is the hypersensitivity to oxidizing agents. The CS cells undergo apoptosis when challenged with low doses of reactive oxygen species (ROS), interpreted as a consequence of non-repaired oxidative DNA damage [39,40]. If this is the case, then knockdown of the enzymes that repair oxidative DNA-damage should provoke a CS-phenotype in mice. However, the knockdown of base-excision repair glycosylases [41], even in combination with the protein responsible for most CS cases, i.e., Cockayne syndrome B protein (CSB), does not result in premature aging [42]. 

The CS phenotype in humans is induced by mutations in the Cockayne syndrome B protein (CSB) (70%), the Cockayne syndrome A protein (CSA) (20%), the subunits of the transcription/DNA repair factor TFIIH, XPB and XPD, and XPG and XPF, and two NER proteins. All of these proteins play central roles in the repair of helix-distorting lesions in DNA provoked by UV light. The total failure of this DNA repair pathway results in the cancer-prone skin disease XP that is characterized by the development of different cancers on UV-exposed skin. Early diagnosis and consequent UV protection reduces skin-cancer risk and allows almost normal development of XP children [43]. 

The CS cases that do not suffer from a combination with XP are cancer free, indicating that developmental impairment and premature aging are not caused by non-repaired mutagenic UV lesions, although CS patients are frequently highly UV sensitive. The same type of UV sensitivity can also be found in UV-sensitivity syndrome (UVsS), a rarely diagnosed hypersensitivity of the skin to UV light [44,45]. UVsS can be caused by mutations in the CS factors CSA or CSB, but does not display the severe phenotype of CS. The cells from UVsS patients display the same DNA-repair defect as the CS cells [46], thus the DNA-repair defect cannot be responsible for the severe phenotype of CS patients. One difference between the UVsS and CS cells is that the latter are hypersensitive to oxidizing agents, and thereby undergo apoptosis when challenged with elevated ROS [45]. The hypersensitivity to oxidation also differentiates the combined XP/CS patient cells from the XP patient cells, indicating that this feature might explain the severe developmental and premature aging symptoms found in CS [47]. The NER proteins are involved in the repair of oxidative DNA lesions [48], however, an oxidative DNA-repair pathway that is specific for CS but not for XP has currently not been identified [38]. Either DNA [39], proteins [49] or lipids [48] might be oxidized and cause the chain of events that result in childhood degeneration and premature aging. Therefore, alternative hypotheses for the premature aging phenotype of CS are discussed [38,49]. One striking feature of all CS-proteins is that they are involved in the rate-limiting step of ribosomal biogenesis, transcription by RNA Pol I in the nucleolus [50,51,52,53,54]. 

## 6. CS Factors Are Involved in RNA Pol I Transcription

### 6.1. TFIIH in RNA Pol I Transcription 

Previously, Iben et al. demonstrated that the TFIIH and the CS proteins, XPB and XPD, are co-immunoprecipitated with RNA Pol I and the RNA Pol I-specific initiation complex containing transcription-initiation factor IB (TIF-IB) indicating a role for the TFIIH in rRNA synthesis. Moreover, co-localization studies of the TFIIH with RNA Pol I and TIF-IB in the nucleolus, at the site of active transcription by RNA Pol I, confirmed this hypothesis. By using electron microscopy and immunogold labelling, the authors demonstrated localization of the TFIIH in the dense fibrillar component (DFC), a nucleolar structure in which pre-rRNA is synthesized. The TFIIH depletion impaired in vitro RNA Pol I transcription activity that could be rescued by adding highly-purified TFIIH in a concentration-dependent manner. These results indicated that the TFIIH is required for productive ribosomal RNA synthesis [50]. 

In 2002, Hoogstaten et al. demonstrated, in a series of elegant experiments, that the TFIIH resides in the nucleolus in a transcription-dependent manner [51]. In this study, the authors employed green fluorescent protein-tagged subunits of TFIIH and used photobleaching to determine the functional dynamics of TFIIH in transcription and repair. Injecting antibodies against the TFIIH subunits reduced RNA Pol I transcription activity in cells and the nucleolar localization of TFIIH was dependent on the ongoing RNA Pol I activity. 

In addition to the fact that TFIIH plays an essential role in transcription by RNA Pol I, Assfalg et al. further characterized the function of TFIIH in rDNA transcription. They demonstrated that TFIIH is bound to the initiation complex, including selectivity factor 1(Sl1), upstream binding factor (UBF), and transcription initiation factor IA TIF-IA with RNA Pol I. Furthermore, by analyzing chromatin immunoprecipitation (ChIP), TFIIH was found to bind to the gene-internal regions of the rDNA, indicating functions of the transcription factor beyond initiation of the RNA Pol I transcription. To study the interaction of TFIIH with the rDNA after transcription initiation, an assay called template-immunoprecipitation assay (TIP) was developed. Here, in vitro transcription and chromatin immunoprecipitation are combined, allowing kinetic analysis of DNA-protein interactions. The rDNA template was added to nuclear extracts with the first nucleotides, allowing synchronization by the formation of the initiation complex. By the addition of the missing nucleotides, single-round transcription was initiated and stopped by the crosslinking agent formaldehyde. On subsequent precipitation of TFIIH and the polymerase, the temporal interaction of TFIIH with the rDNA was analyzed. Immediately after transcription initiation, proteins of the initiation complex, including TIF-IA, leave the rDNA, whereas TFIIH and RNA Pol I are associated longer with the template. Immunoprecipitations after the transcription cycle showed that TFIIH and RNA Pol I are in a complex. These experiments indicated that TFIIH might act as an elongation factor that productively interacts with RNA Pol I. The mutated XPB and XPD subunits of TFIIH, as found in CS cells, reduced the affinity of TFIIH for rDNA and decreased the productivity of the transcription by RNA Pol I, implying qualitative dysfunction of TFIIH in the XPB- and XPD-mutated CS cells. Because the RNA Pol I transcription activity accounts for up to 70% of the total transcription in growing cells, a disturbance in the RNA Pol I transcription action might dysregulate ribosomal biogenesis and hence reduce cell growth. This cellular pathology might explain the growth and neurological retardation observed in CS patients [55].

Nonnekens et al. studied TFIIH and CSB in the RNA Pol I transcription activity in the cells of mouse models of CS and the related progeroid disorder trichothiodystrophy (TTD). Using ChIP analysis, the authors demonstrated that TFIIH binds the entire rDNA coding sequence, supporting the notion that TFIIH is involved in the elongation step of the RNA Pol I transcription activity. The coverage of the rDNA by the polymerase itself is reduced in cells from the CSB mutant and the TFIIH/TTD mice, indicating that CSB and TFIIH are necessary for the recruitment of the enzyme. Moreover, the authors discovered a maturation defect in the multistep process of rRNA cleavage in the TTD cells. This could be confirmed in the brain tissue of TTD mice and suggests that when the transcription process is disturbed, the co-transcriptional maturation and assembly of the ribosomal subunits might also be affected [56]. 

### 6.2. CSA and CSB in RNA Pol I Transcription

However, 90% of CS cases are caused by mutations in CSA or CSB. Interestingly, Bradsher et al. detected a co-localization of CSB, TFIIH and RNA Pol I in the nucleolus, indicating the involvement of CSB in RNA Pol I transcription. They confirmed this hypothesis by using an in vitro transcription system, including rDNA template, initiation factors, and RNA Pol I. The addition of purified recombinant CSB enhanced RNA Pol I transcription by approximately 10-times. Furthermore, they detected a reduced rate of new rRNA synthesis in the CSB-mutated CS cells as compared with the same cell line rescued with the wild-type CSB gene. CSB catenates a complex of RNA Pol I and TFIIH that is reduced in the CSB-mutated CS cells as compared with the rescued cell line with the wild-type CSB gene. Moreover, they isolated a complex of CSB, TFIIH and RNA Pol I that contained the CS protein XPG [52]. 

Yuan et al. investigated the hypothesis that the CSB is an activator of RNA Pol I transcription and is recruited by the transcription terminator factor 1 (TTF1), which is a master regulator of rDNA promoter activity [57]. The CSB protein was shown to stimulate RNA Pol I transcription activity independent of its ATPase domain. Moreover, CSB recruits the histone-methyltransferase G9a that methylates H3K9 in the transcribed region of the rDNA, augments the association of RNA Pol I with the rDNA, and increases rDNA transcription. Therefore, rDNA-transcription stimulation by CSB in the context of chromatin is mediated by histone methylation. In addition to the interaction of CSB to TTF1, Xie et al. further found protein-protein interactions of CSB and components of the nucleosome remodeling and deacetylation complex (NuRD) including CHD4 via tandem affinity purification and mass spectrometry analysis. By using ligation-mediated PCR (LM-PCR) they showed interaction of CHD4 to inactivated rRNA genes, whereas RNA polymerase I associated with active rRNA genes. Surprisingly, CSB interacts with both active and inactive rRNA genes, indicating an association of CSB to rRNA genes that are in a poised nucleosomal confirmation. Hence, they proposed a third state of rRNA genes, where transcription factors bind to the rRNA genes but are not transcribed yet. With these results, they suggested that CSB plays a role in switching the poised rRNA gene to the active state [58]. Lebedev et al. characterized the role of CSB in RNA Pol I transcription activity in CSB-mutant patient cells and control cells. The ChIP experiments revealed a binding of CSB to the promotor and gene-internal regions of the rDNA, implying a role for CSB in the elongation step of RNA Pol I transcription activity. Surprisingly, complete CSB deletion in cells of a UV-sensitive patient demonstrated no effect on RNA Pol I transcription activity, indicating that CSB is not absolutely essential for transcription by RNA Pol I. Indeed, truncated CSB in CSB-mutant CS cells repressed RNA Pol I transcription activity during the elongation step of transcription by RNA Pol I. Therefore, they hypothesized that truncated CSB rather than total CSB deletion leads to premature aging [59]. In addition to CSB, Koch et al. localized CSA to the nucleolus. By using real-time PCR analysis, they detected reduced expression of different regions of the 47S rRNA precursor in CSA-mutated CS cells as compared with CSA wild-type gene-reconstituted cells. Therefore, these results indicate a role for CSA in the initiation and elongation steps of RNA Pol I transcription activity. The involvement of CSA in transcription by RNA Pol I was confirmed by ChIP analysis displaying the binding of CSA to the active (unmethylated) fraction of the rDNA promotor. To address whether CSA and RNA Pol I bind to the same rDNA, ChIP-re-ChIP analysis was performed. Both RNA Pol I and CSA were re-ChIPed by the respective other antibody, indicating interaction of CSA to RNA Pol I on the rDNA. By analyzing the kinetics of the CSA binding to the rDNA, CSA was found to recruit CSB, TFIIH, and RNA Pol I to the transcription site. Moreover, CSA-deficient CS cells displayed reduced mature rRNA 18S, indicating disturbed rRNA maturation. The reduced growth of CSA-mutant cells may be caused by of the lowered ribosomal biogenesis identified in this study [54]. 

**Figure 1:**
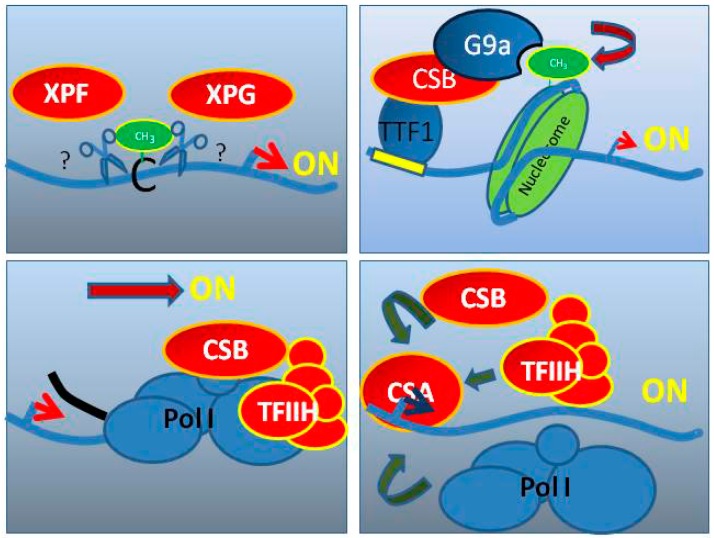
Graphical representation of the presumed/shown functions of the Cockayne syndrome proteins (**red**) in RNA polymerase I transcription. The functions are (**from upper left**), de-methylation of the promoter, attraction of a histone-modifier, reinitiation of basal transcription, and stimulation of elongation.

Schmitz et al. demonstrated that components of the NER pathway, including the CS proteins, XPG and XPF, are required for de-methylation and activation of the rDNA promoter. In the XPG-Cockayne syndrome cells, the number of active rDNA promoters was severely reduced because of hypermethylation and could be rescued by XPG-expression. An XPG mutant protein lacking endonuclease activity was unable to reduce the hypermethylation and inactivation of the rDNA promoters, indicating that the enzymatic activity of XPG is required for the regulation of rDNA transcription [53]. The roles of the CS proteins in RNA Pol I transcription is schematically represented in Figure 1.

## 7. Loss of RNA Pol I Transcription Leads to Mitochondrial Dysfunction

In a series of knockdown experiments, Scheibye-Knudsen et al. demonstrated that there is a close association between ribosomal transcription by RNA Pol I and mitochondrial function [60]. Loss of the CS proteins, CSA or CSB, inhibited rRNA transcription in neuroblastoma cell lines and subsequent microarray analyses revealed a pronounced upregulation of translational and mitochondrial pathways. The specific inhibition of RNA Pol I transcription activity in different cell lines markedly raised mitochondrial membrane potential and superoxide production dependent on poly-ADP ribose polymerase 1 (PARP1). Ruling out that the mitochondrial phenotype is a consequence of translational deficiencies, the authors showed that transcriptional stalling at G4 quadruplex structures is enhanced in the absence of CSA or CSB and that CSB is able to resolve these transcription-obstacles. Interestingly, CSB overexpression in CSA-mutant patient cells could overcome mitochondrial phenotype and PARP1 activation. By avoiding replication effects through the usage of non-dividing cells, the authors demonstrated that treatment with the G4-stabilizing drug pyridostatin, or the RNA Pol I inhibitor CX5461, led to the PARP1 activation. This was followed by the loss of NAD^+^ and an increase of nicotinamide. Reassessing this chain of events in *C. elegans*, the authors demonstrated that inhibition of RNA Pol I transcription activity and stabilization of G4 quadruplex structures lead to accelerated aging and a shortened lifespan that could partially be rescued by a NAD^+^ precursor. Therefore, premature aging in CS is described as a consequence of a failure in the resolution of secondary DNA structures that are predominantly localized in the rDNA.

## 8. Loss of Proteostasis in CS

**Figure 2:**
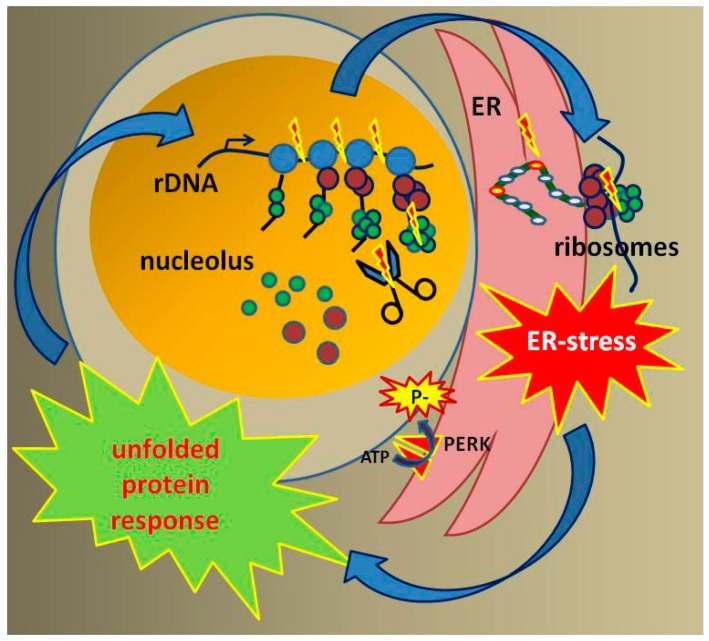
Graphical illustration of the hypothesis: Disturbances in RNA polymerase I transcription result in rRNA processing defects, altered subunit composition, and reduced translational fidelity of the ribosomes. Misfolded proteins provoke ER stress and an UPR that affects ribosomal biogenesis.

In considering the cellular consequences of a disturbance in RNA Pol I transcription, as described in the publications above, Alupei et al. investigated ribosomal function and cellular signaling in CS-patient cells in comparison to reconstituted and UVsS cells [49]. The authors revealed a circulus vitiosus originating from a disturbed ribosomal transcription leading to the repression of this vital pathway as depicted in Figure 2. A reduced rate of the 47S pre-rRNA transcription, indicating disturbed RNA Pol I transcription, and a decreased protein synthesis were detected in CSA- and CSB-mutated CS cells. Interestingly the number of ribosomes was not reduced, suggesting a disturbance in the translation process. Indeed, they found increased translation infidelity in both CSA- and CSB-mutated cells, indicating a malfunction of the ribosomes. The translation accuracy of the ribosome was analyzed by transfection experiments using a mutant luciferase plasmid with a point mutation inactivating the enzyme [61]. With erroneous incorporation of the correct amino acid, the luciferase activity was restored, whereas with accurate translation, the point mutation was translated, and the enzyme remained inactive. Increased luminescence, and thus increased translational infidelity, were observed in CSA- and CSB-mutated CS cells. By protein unfolding using urea and labelling the exposed hydrophobic residue with the fluorescent dye 4,4′-Dianilino-1,1′-binaphthyl-5, (BisANS), the stability of the proteome was determined. The authors observed increased proteome instability in both CSA- and CSB-mutated CS cells. In the presence of elevated ROS levels, an increase of carbonylated proteins in both CSA- and CSB-mutated cells was observed. Moreover, elevated levels of unstable and misfolded proteins led to endoplasmic reticulum (ER) stress, as indicated by an increased protein level of the ER-stress marker GRP78. The ER stress activates the unfolded proteins response (UPR), as observed by increased phosphorylation of eIF2α. The eIF2α phosphorylation leads to apoptosis in both CSA- and CSB-mutated cells. The oxidative hypersensitivity of CS cells was overcome by the addition of different chaperones, indicating that this particular feature of CS cells might not be due to oxidative DNA damage, but rather because of misfolded proteins susceptible to oxidation. Furthermore, it was shown that decreased RNA Pol I transcription activity is not only caused by the mutation of CSA and CSB, but also by ER stress, because treatment with the chaperone tauroursodeoxycholic acid (TUDCA) de-repressed the transcription by RNA Pol I and protein synthesis. Therefore, decreased RNA Pol I transcription is followed by ribosomal malfunction, loss of proteostasis, and ER stress-induced inhibition of rRNA synthesis, which together lead to a vicious cycle and cell death in CS cells. This pathomechanism might explain developmental defects and neurological degeneration observed in CS [49].

## 9. Outlook

The nucleolus and ribosomal transcription deserve greater attention by researchers interested in aging mechanisms. The concept that ribosomal transcription is a process that is unaffected by the functional decline characterizing all cellular and organismal pathways is challenged by the results reviewed in the present manuscript. Whereas, the level of nucleolar activity strongly correlates with the lifespan of model organisms and humans, the quality of transcription by RNA Pol I and the influence on aging is an upcoming issue. The quality of ribosomal performance as a driver of the aging processes was discussed in the early 1960s by L.E. Orgel, who proposed that errors in the translation process would lead to further errors when the ribosomal proteins themselves are affected, a self-perpetuating effect [62]. This “error catastrophe theory of aging” was opposed in the final decade of the 20th century [63], but was never clearly experimentally refuted. Moreover, some researchers found a loss of accuracy of the translation process in aging human fibroblasts [64], a result that could not be confirmed in short-living rodent models [65], indicating that this is not a universal aging hallmark. However, the accuracy of the translation process differs between short- and long-living organisms [66], showing that in fact, long-living species like humans rely on a high quality of protein synthesis. Recently, a study identified decoding-defective ribosomes as a pathomechanism in a human dysmorphic syndrome with microcephaly and impaired intelligence [67]. The fact that humans rely on a high quality of protein synthesis is also supported by the observation that age-associated disorders like Alzheimer’s and Parkinson’s dementia are proteinopathies. These diseases are accompanied or even caused by protein aggregation of an unknown origin. It is tempting to speculate that protein misfolding due to a weakening of translational accuracy in the aging process may contribute to the pathomechanisms of neurodegenerative diseases.
